# Syndemics of syphilis, HCV infection, and methamphetamine use along the east coast of China

**DOI:** 10.1186/1471-2458-14-172

**Published:** 2014-02-17

**Authors:** Meizhen Liao, Dianmin Kang, Xiaorun Tao, Catherine Cox, Yuesheng Qian, Guoyong Wang, Cui Yang, XiaoYan Zhu, Na Zhang, Zhenqiang Bi, Yujiang Jia

**Affiliations:** 1 Institution for AIDS/STD Control and Prevention & Shandong Key Laboratory for Epidemic Disease Control and Prevention, Shandong Center for Disease Control and Prevention, Jinan, Shandong Province 250014, P R China; 2 Department of Epidemiology and Biostatistics, School of Public Health, University of Maryland College Park, College Park, MD 20742, USA; 3 Department of Health, Behavior and Society, Johns Hopkins Bloomberg School of Public Health, Baltimore, MD 21205, USA; 4 Department of Preventive Medicine, Vanderbilt University, Nashville, TN 37232, USA

**Keywords:** Syphilis, HCV, Methamphetamine, Sexual behavior, Drug user

## Abstract

**Background:**

An upsurge in club drug use has been observed in recent years in some cities of China, especially methamphetamine, which is quickly replacing heroin to become the most widespread drug across the nation. This study investigated the type of drugs used, syphilis and hepatitis C virus (HCV) infection and the correlates for syphilis, HCV and unprotected commercial sex behavior among drug users in two cities along the east coast of China.

**Methods:**

A cross-sectional survey conducted in 2010 provided demographics, sexual and drug use behaviors, HIV knowledge and the utilization of intervention services among drug users. Blood samples were tested for HIV, syphilis, and HCV infection.

**Results:**

Of 805 eligible participants, 0.2% were infected with HIV, 3.7% with HCV, and 9.6% with syphilis. Of the participants, 96.6% were methamphetamine users, 11.9% reported ever having used ≥2 types of these drugs, and 11.4% reported ever injecting drugs. In the multivariable logistic regression analysis, participants infected with syphilis were more likely to be female (adjusted odds ratio (AOR)=2.8, 95% confidence interval (CI): 1.2-6.5), have ever had commercial sex in the past 12 months (AOR=2.0, 95% CI: 1.0-3.9), be infected with HCV (AOR=12.1, 95% CI: 4.1-20.3) and less likely to have ever had sex with regular partners in the past 12 months (AOR=0.2, 95% CI: 0.1-0.6). Participants infected with HCV were more likely to have ever injected drugs (AOR=2.7, 95% CI: 1.1-6.5) and be infected with syphilis (AOR=8.0, 95% CI: 3.5-18.0). Participants who had unprotected sex with commercial sex partners in the last sexual encounter were more likely to be female (AOR=2.9, 95% CI:1.7-4.9), have middle school or lower level education (AOR=3.4, 95% CI:2.0-5.5), never have received intervention in the last year (AOR=2.1, 95%CI:1.2-3.6) and be infected with syphilis (AOR=4.2, 95% CI:2.4-7.4).

**Conclusions:**

Methamphetamine is the predominant drug used among the drug users, the prevalence of syphilis and HCV infection are alarmingly high, and unprotected commercial sex was common among this group. The findings highlight the need for effective, multifaceted interventions addressing sexual and drug use-related risky behaviors among this group. Further research is needed to better understand the causal pathway of the syndemics.

## Background

Historically, injection drug use, mainly heroin, was concentrated in the rural areas of southwestern and northwestern China, and has been the major contributor to the HIV/AIDS epidemic in this country [1-3]. However, in recent years, China has seen an upsurge in club drug use, especially methamphetamine, which is quickly replacing heroin to become the most widespread illicit drug across the nation [4-7]. Shandong is one of the provinces confronting the rapidly emerging club drug use. One of the cities along the east coast has been named the “ice city”, for its common use of club drugs, especially among young people. MA and other club drugs are often used in entertainment venue settings, such as hotels, Bars, KTV and tea houses. Club drugs were perceived as a pastime, an entertainment additive without any stigma attached to it, rather than the highly stigmatized traditional substance abuse. Methamphetamine can be smoked, snorted, injected or orally ingested. These drugs meet the psychological characteristics of young people, e.g., curiosity, wonder, and excitement [8,9]. Synthetic drug use fuels the transmission of HIV/other STDs (sexually transmitted diseases) [10,11]. Empirical studies from other countries have demonstrated an association between synthetic drug use and risky sexual behaviors that include unprotected anal and vaginal intercourse, and sexual activities with multiple partners, as well as an association between club drug use and HIV/other STDs [12-15]. Among the 780,000 estimated HIV/AIDS cases in 2011, 63.9% were infected through sexual transmission, and 17.4% through male-to-male contact, which has emerged as one of the major transmission routes in China [16]. New types of drugs, termed synthetic drugs or club drug users, could play a significant role in fueling the epidemics of HIV and other STDs without an effective action taken in a timely fashion [7,17].

China faces the emerging syndemics of HIV, other STDs, and synthetic drugs [18,19]. Studies have been conducted focusing on HIV and other STDs, however, the research on club drugs and its impact on HIV and other STDs remains scarce. Both the extent of the epidemic of club drug use and its social consequences are not yet fully recognized [17]. Limited information is available on the syndemics of HIV/other STDs, Hepatitis C Virus (HCV), and club drugs in China [20]. This study investigated the type of drugs, syphilis and hepatitis C virus (HCV) infection and the correlates for syphilis, HCV and unprotected commercial sex behavior among drug users in Jining City and Qingdao City, Shandong Province of China.

## Methods

### Recruitments and participants

A cross-sectional survey was conducted in Jining and Qingdao, Shandong Province in 2010. Prior to the recruitment of the participants of the survey, we conducted informative research including in-depth interviews with key informants to gather the background information among drug users, e.g., the venues to access them, selection of the candidates of the first group for interview, and sampling frame. All potential participants were invited for an eligibility assessment. Recruitment criteria required that participants be willing to complete the study, and self-report having used drugs in the last year. Participants were approached from drug user gathering venues such as bars, night clubs, bathhouses, saunas, public parks, outdoor cruising areas, and HIV testing sites. After these initial participants were approached and interviewed after an eligibility assessment, we asked the participants to refer their peers to attend the study. A mixed recruitment method, including community outreach, venue-based recruitment, and peer referrals, was applied in the study. Structured questionnaire based interviews were conducted by trained health professionals in a carefully selected clinic in each city. Voluntary participation, anonymity and confidentiality were ensured for all participants. The trained interviewer also conducted some of the interviews within the community or venues such as hotels or bars because some of the participants were not willing to visit the clinics. The experienced study staff explained the study purpose and protocol including voluntary and anonymity of participation, confidentiality and security of data collection, and safety of blood sample collection to all potential participants. An informed consent was received from all eligible participants. The study was approved by the Institutional Review Board (IRB) of Shandong Center for Disease Control and Prevention.

### Measures

Questionnaire-based interviews provided information including demographics (age, marital status, ethnicity, residency, education), sexual and drug use behaviors (sex behavior with regular and commercial partners, condom use with clients or regular sex partners, types of drug use and injection history), HIV knowledge, HIV testing, and HIV-related prevention services. Participants who reported using various types of drugs in the last year were defined as methamphetamine, heroin, cocaine, cannabis, morphine, pethidine, ketamine, MAMD (Ecstasy), and amphetamine users. The regular sex partner refers to their spouse if married or the sex partners that are not married but live together.

Level of HIV knowledge was assessed using eight questions regarding the modes of HIV transmission and prevention, as well as misperception. All questions were weighted equally. Responses to these questions were combined into an overall score for each of the correct and incorrect answers. These scores were then stratified into two groups, according to whether six or more appropriate responses were given to the eight questions. The use of HIV-related prevention services was assessed by whether participants had ever received one of the five indicators related to relevant prevention services in the past 12 months (peer education, HIV counseling and testing, community drug maintenance treatment, needle and syringe provision & exchange, or received free condoms).

Blood samples were collected and tested for HIV, syphilis and HCV. Two screening tests were used to diagnose HIV: an enzyme-linked immunosorbent assay and a confirmatory test using the HIV-1/2 Western Blot immune assay (HIV Blot 2.2 WB; Genelabs Diagnostics, Singapore). Syphilis was diagnosed using rapid plasma reagin (RPR^TM^) and a Passive Particle Agglutination Test for Detection of Antibodies to Treponema pallidum (TPPA^TM^, Rong Sheng Biostix Inc, Shanghai, China). Two screening tests using an enzyme-linked immunoassay (Beijing Wantai Biological Medicine Company, China and Xiamen Xinchuang Industrial Scientific, China) were performed to determine HCV status. All of the participants were informed of their testing results of HIV, syphilis and HCV by trained health professionals. All of the notification of testing results and referrals for positive individuals to subsequent prevention, counseling, and treatment services were also provided in a confidential manner using a prior signed unique code.

### Statistical analyses

Questionnaire-based data and biological results were recorded into the EpiData software (EpiData 3.0 for Windows, the EpiDate Association Odense, Denmark). All analyses were performed with the SPSS® software (Version 15.0; SPSS Inc., Chicago, Illinois, USA). The prevalence rates of syphilis, HCV and unprotected sex were calculated by demographic characteristics, drug use and sexual behaviors, and utilization of HIV-related prevention services. Univariate analyses were performed for the independent variables of demographic, behavioral, and biological variables. A multivariable logistic regression model was constructed. Both adjusted odds ratios (AOR) and 95% confidence intervals (CIs) were calculated for each explanatory variable in the final models. All potential co-linearity, potential confounding, and effect modification were examined and taken into consideration in the final models. Statistical significance for the variables was determined with p < 0.05 in the model.

## Results

### Characteristics of participants

Of 805 eligible participants, 82.8% were male; 28.0% single, 70.2% currently married or cohabitating; the median age was 30 years with a range from 16 to 67 years; 86.5% were local residents; 64.2% received middle school or a lower level of education; only 3.6% belonged to non-Han ethnic groups; 66.8% correctly answered at least 6 out of 8 questions on HIV transmission, prevention and misperceptions; 16.1% received HIV-related intervention in the past 12 months; 4.7% had a test for HIV in the past 12 months while 52.6% knew their HIV test result (Table 1).

**Table 1:** Demographic characteristics, drug use and sexual behaviors, and utilization of HIV-related prevention services among club drug users in Shandong Province, China

**Factors**	**Participants**		**Syphilis**		**HCV**		Unprotected sex*	
	**N=805**	**%**	**n=77**	**%**	**n=30**	**%**	**N=211**	**%**
**Age (years;**$\bar{x}\pm \mathrm{SD})$	30 ± 9.1		32.1 ± 11.4		29.8 ± 10.1		29.9 ± 9.2	
≥18	285	35.4	27	9.5	11	3.9	72	49.0
25-35	281	34.9	20	7.1	11	3.9	62	44.9
35-45	183	22.7	21	11.5	6	3.3	57	54.3
≥45	56	7.0	9	16.1	2	3.6	20	60.6
**Gender**								
Male	662	82.2	52	7.9	26	3.9	176	37.6
Female	143	17.8	25	17.5	4	2.8	35	53.3
**Marital status**								
Single	225	28.0	21	9.3	9	4.0	67	47.2
Married or cohabiting	565	70.2	52	9.2	20	3.5	139	51.5
Divorced or widowed	15	1.8	4	26.7	1	6.7	5	45.5
**Education**								
Middle school or lower	517	64.2	49	9.5	15	2.9	179	58.5
High school or higher	288	35.8	28	9.7	15	5.2	32	27.4
**Types of drugs**^**#**^								
Heroin	26	3.2	0	0.0	3	11.5	2	28.6
Cocaine	1	0.1	0	0.0	0	0.0	1	100
Opium	0	0	0	0.0	0	0.0	0	0
Cannabis	4	0.5	0	0.0	0	0.0	1	33.3
Morphine	1	0.1	0	0.0	0	0.0	0	0
Methamphetamine	778	96.6	77	9.9	28	3.6	207	49.9
Pethidine	0	0	0	0.0	0	0.0	0	0
Ketamine	51	6.3	1	2.0	2	3.9	2	28.6
MAMD	36	4.5	0	0.0	0	0.0	1	20.0
Amphetamine	32	4.0	1	3.1	3	9.4	2	40.0
**Multi-drug use (types)**								
<2	709	88.1	75	10.6	25	3.5	208	51.0
≥2	96	11.9	2	2.1	5	5.2	3	20.0
**Had ever injected**								
Yes	92	11.4	8	8.7	9	9.8	11	44.0
No	713	88.6	69	9.7	21	2.9	200	50.3
**Had ever sex with regular sex partners in last year**								
Yes	558	98.8	49	8.8	20	3.6	138	51.9
No	7	1.2	3	42.9	0	0.0	1	25.0
**Condom use with regular sex partners in last year**								
Always	21	3.8	0	0.0	1	4.8	0	0
Sometimes	120	21.5	6	5.0	2	1.7	4	11.4
Never	417	74.7	43	10.3	17	4.1	134	58.8
**Condom use with regular sex partners in last sex**								
Yes	41	7.3	1	2.4	1	2.4	0	0
No	517	92.7	49	8.8	19	3.7	138	54.5
**Had ever sex with commercial sex partners in last year**								
Yes	423	52.5	49	11.6	13	3.1	-	-
No	382	47.5	28	7.3	17	4.5	-	-
**Condom use with commercial sex partners in last year**								
Always	43	10.1	2	4.7	1	2.3	-	-
Sometimes	169	40.0	23	13.6	3	1.8	-	-
Never	211	49.9	24	11.4	3	1.4	-	-
**Condom use with commercial sex partners in last sex**								
Yes	105	24.8	6	5.7	3	2.9	-	-
No	318	75.2	43	13.5	4	1.3	-	-
**Ever received intervention**								
Yes	130	16.1	14	10.8	5	3.8	29	34.1
No	675	83.9	63	9.3	25	3.7	182	53.8
**Syphilis/HCV infection**								
Syphilis Positive	77	9.6	-	-	11	14.3	24	56.2
HCV Positive	30	3.7	11	36.7	-	-	3	42.9
Syphilis/HCV Negative	709	88.1	-	-	-	-	186	26.2
**HIV knowledge score**								
Score<6	267	33.2	31	11.6	7	2.6	155	72.1
Score ≥ 6	538	66.8	46	8.6	23	4.3	56	26.9

Subtotals do not add up to 100% for some variables because of missing values.

*Unprotected sex means no condom use with commercial sex partners in the last year.

^**#**^The factor of “types of drugs” was multi-choice variables, the percentages will not add up to 100%.

### Drug use, sexual behaviors and correlates for unprotected sex

Of the participants, 96.6% (n=778) were methamphetamine users, 6.3% (n=51) ketamine users, 4.5% (n=36) MAMD users, 4.0% (n=32) amphetamine users and 3.2% (n=26) heroin users. Of the participants, 11.9% (n=96) reported having ever used ≥2 types of these drugs and 11.4% (n=92) reported having ever injected drugs. Of the participants, 98.8% reported ever having sex with regular sex partners, with 74.7% never having used condoms in the past 12 months and 92.7% reporting no condom use in the last sexual encounter. 52.5% of participants reported having commercial sex, with 89.9% reporting inconsistent condom use in the past 12 months and 75.2% reporting no condom use in the last sexual encounter (Table 1). Participants who have ever had unprotected sex with commercial sex partners in the last sexual encounter were more likely to be female, have received middle school or a lower level of education, have never received an intervention in the last year and be infected with syphilis (Table 2).

**Table 2:** Factors associated with syphilis and HCV infection among club drug users in Shandong, China

**Factors**	**Syphilis infection(n**=**77)**			**HCV infection (n**=**30)**			**Unprotected sex**		
	**%**	**OR(95% CI)**	**AOR(95% CI)**	**%**	**OR(95% CI)**	**AOR(95% CI)**	**%**	**OR(95% CI)**	**AOR(95% CI)**
**Gender**									
Male	7.9	1.0	1.0	3.9	1.0		37.6	1.0	1.0
Female	17.5	2.5(1.5-4.2)**	2.8(1.2-6.5)*	2.8	0.7(0.2-2.0)		53.3	3.0(1.8-5.1)**	2.9(1.7-4.9)**
**Education**									
Middle school or lower	9.5	1.0		2.9	1.0		58.5	3.7(2.3-5.9)**	3.4(2.0-5.5)**
High school or higher	9.7	1.0(0.6-1.7)		5.2	1.8(0.9-3.8)		27.4	1.0	1.0
**Had ever injected**									
Yes	8.7	0.9(0.4-1.9)		9.8	3.6(1.6-8.1)**	2.7(1.1-6.5)*	44.0	0.8(0.4-1.7)	
No	9.7	1.0		2.9	1.0	1.0	50.3	1.0	
**Had ever sex with regular sex partners in last year**									
Yes	8.8	0.1(0.1-0.6)**	0.2(0.1-0.6)**	3.6					
No	42.9	1.0		0.0					
**Had ever sex with commercial sex partners in last year**									
Yes	11.6	1.7(1.0-2.7)*	2.0(1.0-3.9)*	3.1	0.7 (0.5-1.2)				
No	7.3	1.0	1.0	4.5	1.0				
**Ever received intervention**									
Yes	10.8	1.0		3.8	1.0		34.1	1.0	1.0
No	9.3	0.9(0.5-1.6)		3.7	1(0.4-2.6)		53.8	2.3(1.4-3.7)**	2.1(1.2-3.6)*
**HCV infection**									
Yes	36.7	6.2(2.8-13.6)**	12.1(4.1-20.3)**				42.9	0.9(0.4-1.9)	
No	8.5	1.0	1.0				45.4	1.0	
**Syphilis**									
Yes				14.3	6.2(2.8-13.6)**	8.0(3.5-18.0)**	56.2	3.0(1.8-5.1)**	4.2(2.4-7.4)**
No				2.6	1.0		27.9	1.0	1.0

OR: Odds ratio; AOR: Adjusted odds ratio, controlled factors included study sites, age, sex, residency, ethnicity, education, marital status, sexual behavior, HIV-related prevention services; CI: Confidence interval; NA: Not applicable; * P < 0.05; ** P < 0.01.

### Correlates for syphilis, HCV infection

Of the participants, 0.2% (n=2) were infected with HIV, 9.6% (n=77) with syphilis and 3.7% (n=30) with HCV. 1.4% (n=11) were infected with both syphilis and HCV. All syphilis cases (n=77) were methamphetamine users with 2 of them also ketamine and amphetamine users (Table 1). The participants infected with syphilis were more likely to be female, have ever had commercial sex in the past 12 months, be infected with HCV and less likely to have ever had sex with regular sex partners in the past 12 months. Participants infected with HCV were more likely to have ever injected drugs and be co-infected with syphilis (Table 2).

## Discussion

This is the first to report on the assessment of the syndemics of HIV, syphilis, HCV, and MA use among drug users in China. This study found alarmingly high rates of syphilis (9.6%), HCV infection (3.7%), and MA use (96.6%) among drug users in the two cities along the east coast of China with historically low HIV prevalence. Syphilis and HCV infection predicted each other, and being female was a common correlate for syphilis and unprotected sex. The findings of this study contribute to the understanding of the emergent role of club drug abuse on the HIV/syphilis epidemic and its association with risky sexual behavior in the two cities of Shandong Province.

This study showed that nearly 10% of participants were infected with syphilis, and all of them were MA users. The syphilis prevalence among drug users was significantly higher than that of female sex workers (3.07%), men who have sex with men (4.69%), pregnant women (0.33%) and unpaid blood donors (0.08%) in the same year. The logistic analysis revealed that syphilis was independently associated with ever having had unprotected sex with commercial sex partners in the past 12 months. Moreover, an alarmingly high prevalence (52.5%) of drug users self-reported ever having had sex with commercial sex partners, with 89.9% of these participants self-reporting inconsistent condom use in this study. MA use enhances sexual performance, sensitivity, and pleasure, resulting in a complexity of sexual partners and network, thereby increasing the risk of trauma from prolonged intercourse and failure to use condoms due to altered judgment and inhibition [21,22]. It is even more worrisome that club drug users will take a potential role in the epidemiologic bridge of HIV/syphilis transmission to their regular sex partners [22]. This study also showed that club drug users had a low condom use rate with their regular sex partners. Trust in a sex partner or worrying suspicion ever having had commercial sex behavior by their regular sex partner resulted in low condom use with their regular sex partners. This indicates a great risk in transmission and/or acquisition of HIV/STDs within the club drug abuse and sex networks.

Results also showed participants who have ever had unprotected sex with commercial sex partners in the last sexual encounter were more likely to be female and the participants infected with syphilis were more likely to be female. Being female is the most vulnerable group for possible syphilis infection. In this study, of all participants, 17.8% (n=143) were female, among which 65.0% (n=93) reported having commercial sex. The syphilis prevalence was significantly higher among those females who have ever had commercial sex than those females who have never had commercial sex (22.5% VS. 8.0%). Our previous studies have shown the prevalence of syphilis infection increasing because of the club drug abuse among female sex workers (FSWs) in Qingdao [7]. However, the current intervention efforts are still focused on heroin, which played an important role in China’s HIV/AIDS epidemic in the last decades, and the prevention infrastructure has not been strategized to tackle the newly emerging challenge of club drug abuse. In addition, this study also showed that only 4.7% of participants ever had a test for HIV while 52.6% knew their HIV test result, and 16.1% had received intervention in the past 12 months. A low number of HIV tests and HIV-related prevention services reflected the less intervention efforts targeted this group [1,23], and the low practice of VCT still remains a challenge among targeted drug users. Unprotected heterosexual contacts has been increasing proportionally in contribution of HIV transmission in China, therefore it is possible that the club drug abuse could further fuel this trend in the nation if no better-targeted, effective intervention efforts are taken in a timely fashion [24].

Independent risk factors for HCV were injection drug users and co-infection with syphilis. According to this study, needle sharing of injection drug users could be a reasonable cause of the HCV infections. The findings underscore that harm reduction clear needle should be emphasized. Meanwhile, the high prevalence of syphilis demonstrated that a large number of drug users engaged in unprotected sex behaviors that may place them at high risk for HCV. HCV could be transmitted through parenteral exposures to contaminated blood, with efficiency through the use of injection drugs, e.g., sharing of needles or works [2,3]. Studies have revealed that HCV risk increases commensurate with increasing numbers of heterosexual sex partners [25]. Studies have found that common factors for HCV were associated with group sex, accompanied by STDs, the use of club drugs and other non-injection drugs during sex [26,27]. More research is needed to better understand the role of heterosexual transmission of HCV among this emerging group with a high risk for HIV and club drug use.

This study revealed that the prevalence of syphilis/HCV infection is alarmingly high and unprotected commercial sex was common among drug users. New types of drugs, especially MA, have become the predominant sources of drug abuse in these two cities in China. The findings of this study underscored the urgent need for comprehensive intervention strategies, including sex and drug-related interventions and HIV/syphilis/HCV control among this group. Methamphetamine is the predominant drug used among the drug users. We also should pay more attention to the intervention efforts to target syphilis prevention in FSWs, especially the drug using FSWs, and further research is needed to better understand the causal pathway of the syndemics, the driving forces for the high prevalence of syphilis, HCV, and MA use.

We recognized the limitations of this study. Data collection relying on retrospective self-reports was subject to recall bias. The sensitive nature of the questions (related to sexual and drug use behaviors) may have led to information bias due to the social desirability of certain answers. We didn’t collect participants’ sexual preference in this study. The cross-sectional nature of the data also makes it impossible to establish any causality. It may be another limitation that the non-response or refusal of participation during the recruitment process could reduce the representativeness of the study participants. Non-response could be substantial in this study because drug use is illegal in China. Information on study participation was lacking. Moreover, the recruitment criterion to complete the study may lead to a selection bias. Therefore, the results of this study might not fully generalizable to drug users in other settings in China or different countries. Despite these limitations and possible biases, we feel the data highlights a prevention opportunity that cannot be ignored.

## Conclusions

Synthetic drugs, especially methamphetamine, is the predominant drug used among the drug users in these two cities in China, the prevalence of syphilis and HCV infection are alarmingly high, and unprotected commercial sex was common among this group. The use of methamphetamine could fuel the epidemics of HIV, syphilis and other STDs in China. So far, no effective intervention programs are available targeting synthetic drug users in China. The findings highlight the need for effective, multifaceted interventions addressing sexual and drug use-related risky behaviors among this group. Further research is needed to better understand the causal pathway of the syndemics.

## Abbreviations

HCV: Hepatitis C virus; HIV: Human immunodeficiency virus; MA: Methamphetamine; STD: Sexually transmitted disease; AIDS: Acquired immunodeficiency syndrome; CI: Confidence interval; AOR: Adjusted odds ratio.

## Competing interests

The authors declare that they have no competing interests.

## Authors’ contributions

ML, DK, XT, KY, YQ, GW, XZ, NZ and ZB directed the study data collection and managed the data. ML, CC, CY, XZ, NZ and YJ conducted the primary analyses and developed the first draft of the manuscript. YJ, ML, and DK provided interpretation to the analyses and wrote parts of the methods, results or discussion section or provided major editing. All authors contributed to the writing process before the final version was approved. All authors read and approved the final manuscript.

## Pre-publication history

The pre-publication history for this paper can be accessed here:

http://www.biomedcentral.com/1471-2458/14/172/prepub
